# Pneumonia classification: A limited data approach for global understanding

**DOI:** 10.1016/j.heliyon.2024.e26177

**Published:** 2024-02-14

**Authors:** Anwar ul Haque, Sayeed Ghani, Muhammad Saeed, Hardy Schloer

**Affiliations:** a SMCS, Institute of Business Administration Karachi, Pakistan; b Department of Computer Science, University of Karachi, Pakistan; c Alpha Centauri International, UAE

**Keywords:** Capsule networks clusters, Deep learning, Dicom, Pneumonia, X-rays

## Abstract

As the human race has advanced, so too have the ailments that afflict it. Diseases such as pneumonia, once considered to be basic flu or allergies, have evolved into more severe forms, including SARs and COVID-19, presenting significant risks to people worldwide. In our study, we focused on categorizing pneumonia-related inflammation in chest X-rays (CXR) using a relatively small dataset. Our approach was to encompass a comprehensive view, addressing every potential area of inflammation in the CXR. We employed enhanced class activation maps (mCAM) to meet the clinical criteria for classification rationale. Our model incorporates capsule network clusters (CNsC), which aids in learning different aspects such as geometry, orientation, and position of the inflammation seen in the CXR. Our Capsule Network Clusters (CNsC) rapidly interpret various perspectives in a single CXR without needing image augmentation, a common necessity in existing detection models. This approach significantly cuts down on training and evaluation durations. We conducted thorough testing using the RSNA pneumonia dataset of CXR images, achieving accuracy and recall rates as high as 98.3% and 99.5% in our conclusive tests. Additionally, we observed encouraging outcomes when applying our trained model to standard X-ray images obtained from medical clinics.

## 1 Introduction

Pneumonia, also termed winter fever, dates back to the early ages of human life and was diagnosed by the Greek physician Hippocrates around 460 BCE It was considered a sickness until the 19th century, later identified as an infectious disease. Edwin Klebs (1875) performed a microscopic analysis of pneumonia-causing bacteria, which took a significant leap in curing pulmonary infections. Later, in the 1880s, Carl Friedlander identified two bacterial causes of pneumonia. By the 1930s, antibiotic penicillin medication helped drop the mortality rate. However, the infection rate remained high even with the plethora of ways of cure [[1], [2], [3]]. During the last decade, excluding COVID-19, annual expenses on diagnosis, cure, and treatment of pneumonia were approximately $ 17 billion globally.

Streptococcus pneumonia stands as a primary bacterial culprit in lung infections. This infection notably diminishes survival prospects, particularly impacting the elderly and those with weakened immune systems [4]. The main reason for the spread of viruses like pneumonia is physical contact in workplaces, schools, grocery stores, hospitals, and even nursing homes. The general mechanism of transportation is by breathing the infected air particles. Therefore, the most effective prevention is to avoid breathing space near the infected person or get the vaccine beforehand. The Centers for Disease Control recommends PCV13 [41,42] and PPSV23 [[42], [43], [44]] vaccines for prevention, especially for children under 13 and those seeking international travel.

Pneumonia symptoms are variable, and a conclusive decision requires multiple examinations and evaluations. The tests may include taking a medical history, travel details, exposure summary, and physical analysis like checking the lungs via a stethoscope to find symptoms such as crackling, bubbling, and rumbling sounds during air inhale. Unfortunately, all these tests never provide a compelling conclusion as the outcome is directly proportional to the specialty and understanding of the physician to perceive the problem. Generally, the confusion leads towards more extensive examination like taking blood samples to find the infection and identify germs, chest and back x-rays locating the inflammation in the lungs, measurement of oxygen steam level in blood using a pulse oximeter, mucus sampling during deep cough to find the infectious bacteria, CT scans, Arterial blood gas (ABG) test, pleural fluid culture (PFC), and bronchoscopy [[5], [6], [7], [8]].

The abovementioned methodologies dominate the healthcare industry in finding and curing pneumonia globally and have been a sustaining source of human life-saving practices. However, these procedures rely on human experience and cost much time and money. A patient in the triage requires immediate and life-saving diagnosis and treatment as the extent of severe pneumonia could lead to their demise.

On the scale of diagnosis versus result versus cost, chest X-rays (CXR) are thought to be problem-solving and efficient for pneumonia diagnosis and cure plan description by the doctors. CXR offers doctors the primary means of diagnosis after physical examination and helps increase lung patients' survival rates. However, despite the effectiveness in detecting the lung inflammation caused by pneumonia, CXR requires a trained and experienced radiologist. Given the human faction and workload, misclassification or overclassification is eminent in the day-to-day diagnosis of pneumonia patients. A misclassification leads to life risk, while an over-classification would result in wrong medication and further complexities. Both outcomes could become a disaster for any patient, especially for the elderly and children.

The research community has used several Deep Learning-based methods and solutions to find better pneumonia diagnosis methods using CXR, and the important ones are biomedical imaging and convolutional networks [9]. In computer vision, CNN (Convolutional Neural Network) has always been appreciated and used globally to solve various challenges and tasks. For example, CNN is the benchmark for image classification, object detection, and segmentation problems. However, CNN always requires extensive training data and can easily give incorrect classifications with slight modifications in the object's geometrical, positional, and orientation details under consideration. Therefore, the current standing of deep learning-based pneumonia classification with CNN for clinical decision-making may be significantly improved using a capsule network-based model, which can address the issue of understanding the underlying relationship of an object with its surroundings.

In our work, we aim to aid in diagnosing inflammation due to pneumonia in chest X-rays by providing a lightweight geometrical and positional understanding-based deep learning network. Our proposed network is a composition of a capsule networks cluster [28] and a modified class activation map. The capsule networks cluster-based model is light because it requires very little data to be trained to understand the geometrical, orientational, and positional inflammation details in CXR. The features are extracted in the primary caps layer, while the inflammation caps layer performs the classification. Three fully connected layers evaluate and compare the results with actual results during the training stage. The horizontal scaling in clustering helps us improve performance, response time, and a much deeper understanding of object inherent relationships. The inherent clustering behavior in Capsule Networks is a pivotal attribute contributing to its computational efficiency, rendering the model highly lightweight in terms of computational demands. This property is paramount in medical analysis as lightweight models can be executed on standard workstations, delivering near real-time responses, which is particularly valuable for time-sensitive medical applications. Furthermore, this lightweight characteristic does not compromise the model's ability to comprehend intricate geometrical and orientational details, allowing it to adopt a comprehensive global perspective in its analysis.

Adopting a global perspective holds paramount importance, as it equips the model with the capability to attain a high level of diagnostic accuracy that adheres to rigorous medical standards, all within a single comprehensive training round. This underscores the model's proficiency in delivering reliable and precise diagnostic decisions, extending its capacity to discern pneumonia conditions holistically. This strategic approach to training is particularly beneficial, as it fosters the development of a versatile and adaptable diagnostic model that can operate effectively across various datasets. Utilizing a limited data approach ensures that once trained effectively, the model becomes a robust tool for accurate and efficient pneumonia diagnoses, aligning perfectly with the urgent requirements of healthcare solutions where accuracy and timeliness are essential.

We expect that pneumonia detection having the capability to understand geometrical and orientational relationships would play a vital role in improving healthcare and reducing the overall cost of treatment. Furthermore, a system like this would ensure timely response to patients in critical care and provide doctors and healthcare professionals a means for a speedier response. The main contributions of our work are as follows:

1. Issues of over-fitting and under-fitting within the data have been catered to using the primary capsule-based feature extraction, which treats each object according to its respective geometrical understanding over the original dataset instead of pre-processing, as shown in Fig. 1.

**Fig. 1:**
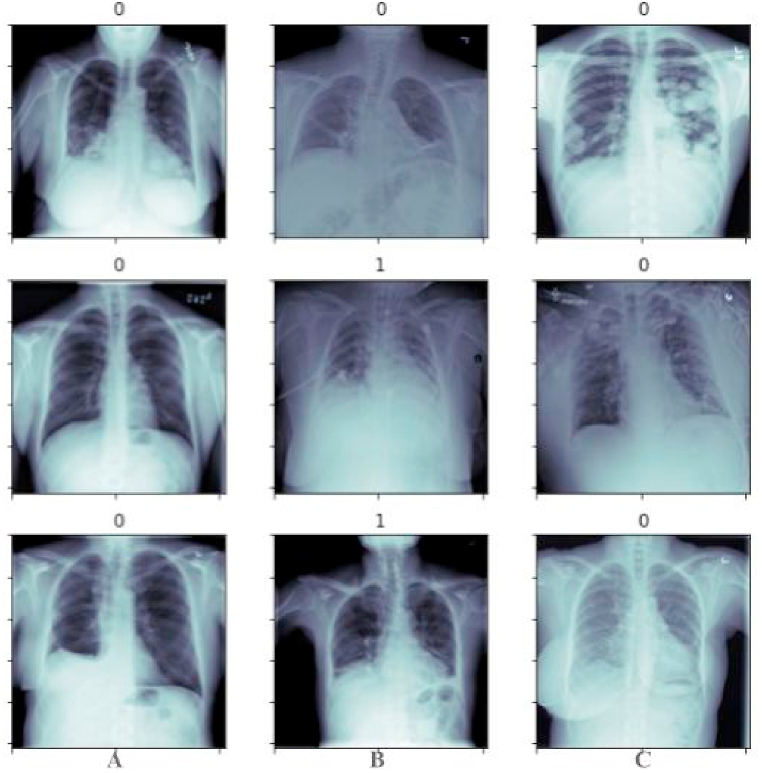
A randomized plot of original images of the RSNA dataset in Dicom format. All images in panels “A”, “B”, and “C” with label “1” represents CXR with inflammation, while all images with label “0” is without inflammation.
2. The essential features of CXR images are considered by modified class activation maps focusing on the region based on feature classes, as shown in Fig. 2.

**Fig. 2:**
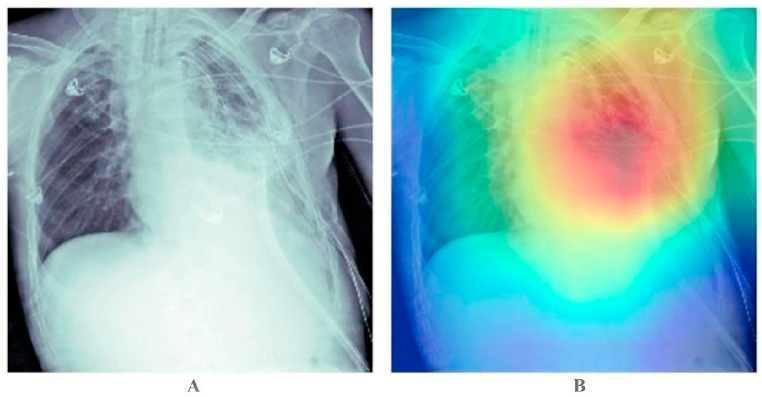
Result of mCAM for our capsule networks cluster to find the region of maximum inflammation within the CXR. Image “A” is original image while image “B” is processed through our model indicating the region of maximum inflammation.
3. Classifying the inflammation using an inflammation caps layer that respects every feature's positional and orientation details, thus ensuring less over-fitting among classes.

The remaining paper is as follows: Section 2, Literature Review, presents related work and other significant contributions in pneumonia classification using deep learning. Section 3, Materials, discusses the preprocessing requirements of the data for our proposed methodology. It also discusses the Dataset and provides a comprehensive discussion of data and its source, types, details, and how we used it for our work. Section 4, Methodology, describes the methods we have adopted in our research, the mathematical foundations of our work, and their modifications to get results as per our desire. Section 5 provides the facts about the proof of concept and the final experimentation and execution of a complete walk-through of the methodology and results and a comparison of our results. Finally, section 6. presents the conclusion and considers future research.

## 2 Literature Review

Regarding CXR analysis for pneumonia detection, we find that this is a long-standing problem in computer vision and healthcare. The reasons for the problem [10,11] are variations in inflammation, types, density of x-rays, and other detection features, which vary from dataset to dataset and x-ray to x-ray. Thus, having a single and straightforward solution to generalize all the issues is still a long-standing wish. Along with other problems related to having a generic solution, a public dataset that can cover various possibilities of occurrences is also the main hindrance. The pneumonia classification started with the machine learning approach, where researchers used handcrafted and manually annotated features for classification [12,13]. However, the entire domain shifted from machine learning to deep learning due to an increasing number of features. The deep learning-based pneumonia classification provided some state-of-the-art works [14,15] to the research community, and even some received appreciation from the healthcare industry.

In [16], the researcher introduced a method that amalgamates the output weights from various models, including ResNet18, InceptionV3, Xception, MobileNetV3, and DenseNet-121, resulting in an impressive accuracy rate of 98.43%. Nonetheless, due to this blend of models, the system is quite resource-intensive and demands significantly more preprocessing than other models. The authors in Ref. [17] evaluated various deep learning models for classifying pneumonia from CXR. They found that customized VGG16 (Visual Geometry Group) performed far better than the rest, achieving 96.2% accuracy in detection while 91.8% in classifying the viral and bacterial types of pneumonia. On the contrary, authors in Ref. [18] found DenseNet201 performing better than the other models over the pneumonia Kaggle dataset. They generated an accuracy of 98% in detection while remaining 93.3% times classifying the images for viral and bacterial pneumonia.

Many researchers have tried novel combinations like in Ref. [19], where CNN and KNN (K Nearest Neighbours) combine. The feature extraction is done with the help of CNN in stage 1, while the later stage performs classification using the K-nearest neighbor of the extracted features. Along with KNN, the authors have also used SVM (Support Vector Machine) to enhance classification accuracy. Machine learning models often depend upon the dataset source, which leads to over-fitting or under-fitting during classification. Authors in Ref. [20] have used a framework with adversarial optimization. Adversarial optimization assists in reducing the model's reliance on the dataset. Yet, the reported AUC (Area Under the Curve) scores, 74.7% in the original domain and 73.9% in target domains, fall short of prevailing benchmarks. The study referenced as [21] explores a confidence-based approach to identifying inflammation, treating it as a single-class issue. It aims to distinguish and isolate CXRs exhibiting atypical patterns from the norm.

Likewise, a distinct approach involving fuzzy tree transformation based on machine learning is presented in Ref. [22]. This method utilizes a multi-kernel binary pattern for feature extraction, employing conventional machine-learning classifiers for categorization. The experimental dataset was small, consisting of simple pneumonia and COVID-19 samples. The quoted result is 97.01%.

Contemporary approaches in pneumonia classification are increasingly centered around diverse and altered deep learning structures, particularly Convolutional Neural Networks (CNNs) as exemplified in Ref. [23]. In this context, the authors introduced a 3-dimensional CNN, which they have named MSH-CNN. The MSH-CNN requires CT scans instead of CXR so that it can perform 3-D analysis of data. The approach seems right; however, getting chest CT scans is another challenging task. In Ref. [24], Mask-RCNN (Region-based Convolutional Neural Network), already state-of-the-art in image classification and object detection, is used. In addition, the work uses dropout and L2 regularization, which helps distinguish the general and specific features of pulmonary scans.

Another approach that multiple authors took is to have augmented data that can provide various combinations during the training stage. For example, in Ref. [25], the authors have performed several preprocessing over the original data, such as random rotation, transition, axis tilting, color masking, representing images in the horizontal and vertical axis, etc. This preprocessing has increased training data size and generated new combinations for the model to learn. However, despite the high accuracy of the quoted result, the method only applies to the healthcare industry as a lightweight classification engine with a global perspective.

Various other methods have proven successful in achieving precise and useful categorizations. For instance, the study referenced as [26] employed transfer learning techniques with two CNN architectures, Xception-net and VGG16. Likewise, the research in Ref. [27] experimented with an amalgamation of results from four advanced learning models: ResNet152, MobileNet, CNN, and LSTM (Long Short-Term Memory). The research [30] uses deep learning, specifically a model based on VGG16, to detect and classify pneumonia in chest X-ray (CXR) images. Since the paper is post-COVID-19, it highlights the need for rapid pneumonia detection to improve treatment outcomes. The research relies on two specific CXR image datasets, which may not fully represent the diversity of pneumonia cases in real-world clinical practice. The model's performance might vary when applied to more varied datasets. The paper does not discuss the clinical validation of the deep learning model. While it shows promising results in accuracy and other assessments, its real-world clinical applicability and impact on patient care are crucial. The paper primarily focuses on the choice of the VGG16 architecture. Further exploration of hyperparameters and model fine-tuning could lead to even better performance.

The results of the Computer-Aided Diagnostics (CAD) scheme in Ref. [31] indicate an overall accuracy of 94.5% in classifying the three categories, with a 95% confidence interval of [0.93, 0.96]. The CAD scheme also demonstrates high sensitivity (98.4%) in detecting COVID-19 cases and high specificity (98.0%) in identifying cases without COVID-19 infection. Using the two preprocessing steps significantly improves the classification accuracy; without them, the accuracy drops to 88.0%. The study's performance is evaluated on a specific dataset, and its generalizability to other datasets or real-world clinical settings may be limited. The performance of the CAD scheme may not be as high when applied to different datasets. The work does not mention the processing time required for the CAD scheme. Real-time diagnosis is essential in clinical settings, and if the system is too slow, it may not be practical for use in emergencies. While the dataset is divided into training, validation, and testing subsets with equal frequencies of cases in each class, the actual prevalence of these categories in clinical practice may not be balanced. The model's performance in cases with imbalanced data might be different.

The paper [32] is one of the best in finding COVID-19 infections using deep learning and CXR. However, the major flaw is the convolutional neural network (CNN). CNN has the inherent issue of being unable to find the covariances and geometrical alignments, which always leads CNN to misread the images [46,47]. From a medical perspective, this is a critical composition while performing diagnosis. Generally, CNN requires huge training data and all possible geometrical and orientational details to be trained for generics, which is impossible to produce. The work [33] introduces a novel CNN architecture for the multi-class classification of X-ray images into pneumonia, healthy, and COVID-19 categories. However, the rationale for selecting DenseNet as the base architecture lacks explanation, and there is a lack of specific details on how the proposed model handles the variability in X-ray images. The modifications, including branched convolutions and instance normalization, are mentioned without in-depth elaboration on their impact. Furthermore, it lacks clarity regarding the evaluation and validation processes, including metrics and comparisons with existing models or radiologist interpretations.

The research [34] provides valuable information about the structure, evaluation metrics, and generalization efforts of the proposed CNNCF (Convolutional Neural Network Classification Framework) framework; however, there are several things to consider. The hyper-parameter values (Q, L, M, N) are mentioned but lack a clear rationale or justification for their selection, which reduces the transparency and reproducibility of the model. While it mentions using multiple experts for evaluation, it does not address potential inter-rater variability or how expert discrepancies were managed, which is critical for the framework's robustness. Furthermore, the methodology does not offer insights into generalizing the proposed CNN model to a broader patient population or multiple healthcare settings. It is vital to address these issues to ensure the model's applicability across different scenarios.

Research [35] introduces an innovative CNN design, STM-RENet, for analyzing radiographic patterns in X-ray images to identify COVID-19. This model distinctively applies a split-transform-merge strategy and integrates region and edge-focused techniques to assess region uniformity, intensity variability, and edge-related characteristics. To boost its learning capabilities, CB-STM-RENet is developed, enhancing the model with channel boosting and texture variation analysis using Transfer Learning. The authors report that CB-STM-RENet surpasses conventional CNNs in performance on three distinct datasets, notably achieving a 97% detection rate, 96.53% accuracy, and a solid F-score of 95% on the CoV-NonCoV-15k dataset, underscoring its efficacy in detecting COVID-19 patients. Despite these claims of superiority over standard CNNs, the study lacks specific comparisons or benchmarks against other models, which would provide a clearer framework for assessing the effectiveness of these models.

The study [36] does not establish the novelty of the proposed approach. While it mentions using a deep learning approach based on the pre-trained AlexNet model, it does not highlight any significant advancements or innovations in the methodology. It would be beneficial to emphasize how this approach differs from existing methods and why it is a notable contribution. The authors mentioned acquiring chest X-ray images from different public databases, but it does not specify the sources or provide information about the dataset's size and diversity. The study is good but requires comparison metrics like Area Under the Curve (AUC), F1, and precision to develop a more generic response and research.

The research paper [37] outlines a study that focuses on using a convolutional neural network (CNN) to detect COVID-19 from chest X-ray (CXR) images. The study discusses the significance of COVID-19 as a global health crisis, highlights its symptoms, and underscores the importance of contact tracing to prevent the virus's spread. The paper employs a convolutional neural network (CNN) approach to detect COVID-19 from chest X-ray images, an innovative and relevant application of deep learning. CNN has been a defacto standard for image-related learning. However, it is one of the brute force techniques that can always go wrong given the changes in the image dynamics, including, but not limited to, lights, surroundings, orientation, geometry, size, etc. So, employing CNN is not one of the techniques generally preferred by the medical community [48,49].

The work [38] discusses a novel approach to weight allocation in ensemble learning, aiming to enhance the model's performance by considering various evaluation metrics beyond accuracy, which is particularly valuable for class-imbalanced datasets. This approach utilizes probability scores from base learners during training to calculate weights based on precision, recall, f1-score, and AUC, introducing a unique strategy for constructing an ensemble. Using a hyperbolic tangent function to determine weights provides a theoretically grounded method for prioritizing base learners. However, concerns about the approach's complexity, potential lack of interpretability, validation, scalability, and generalizability must be addressed to determine its real-world applicability and advantages over traditional weighting methods. While the approach incorporates various metrics, it does not discuss the interpretability of the final ensemble model. Understanding the relative contributions of different base learners is essential for trust and model transparency.

[29] underscores the difficulty distinguishing pneumonia from other respiratory ailments, given their similar visual characteristics and the diverse methods of capturing and processing chest X-ray images. It rightly stresses the necessity for potent, algorithm-based solutions developed using extensive, high-quality data sets, along with the criticality of validation through multiple imaging methods and expert radiological evaluation. Although the research shows encouraging outcomes using eight pre-trained models on two sizeable datasets, a more thorough exploration of its constraints, possible dataset biases, and the compromises involved in selecting hyperparameters would enhance its value. Additionally, combining eight different pre-trained models to acquire a result is a complex issue that lacks practical implementation [45]. The study addresses the global COVID-19 spread and emphasizes accurate diagnostics. The authors explored the CXRs and the detection of widespread with the help of a GAN (Generative adversarial networks)-based approach. The GAN arguments images for custom convolution neural network-based processing. The quoted requests are 99% on the successive hit ratio. However, the study has not tried real-world clinical data to validate their work. The paper [50] proposed a modified capsule network for detecting and classifying pneumonia using X-ray images. The model comprises an encoder with convolutional, primary capsule, and digital capsule layers and a decoder with a deconvolutional layer. Through dynamic routing, the primary and digital capsule layers convert scalars into vectors and cluster vectors of the same category. Training and testing occur on a dataset of 5856 images, achieving a high accuracy rate of 98.6%. The model's streamlined structure and fewer parameters suggest greater ease of deployment in practical applications than other popular models. However, the size of the dataset and the limited training over that nullifies the effectiveness of the model and the work. Study [51] focused on Deep Learning-based diagnosis, specifically using Convolutional Neural Networks (CNNs), for classifying three types of chest X-ray images: normal (1583 images), pneumonia-diagnosed (4273 images), and COVID-19-diagnosed (262 images). Five established architectures (VGG16, VGG19, Xception, InceptionV3, Inception-ResNetV2) are tested on image diagnosis, and these architectures are also used as feature extractors for a capsule layer with 16 dimensions and four routings. VGG16-based capsule network surpasses other architectures with an accuracy of 96.81%. The proposed VGG16-based capsule network architecture is suggested as a potentially fast and accurate alternative for X-ray classification and screening. However, the details of the pre-trained VGG16 for the capsule network are not shared, and the quoted results are well below the state-of-the-art accuracy. Furthermore, the dataset is small and does not generalize for a medical use case. The proposed model in Ref. [52] is trained over CXRs from Guangzhou Women's and Children's Medical Center. The training was done on original and Contrast Limited AHE (CLAHE). They quoted results for accuracy, precision, recall, F1-score, and AUC scores of 0.984, 0.996, 0.971, 0.983, and 0.974, respectively. Applying CLAHE improves study performance, reducing training time by approximately 4%. Chest-Caps outperform the state-of-the-art results as per the paper.

Despite the research mentioned above and many others in the domain of pneumonia classification using deep learning, a lightweight, less data-hungry, and medical-grade accuracy level model still needs to be completed. The current benchmarks need to give generic responses for the problem and always require more and more data. They also seek considerable attention while training them and tuning their hyperparameters.

The current state-of-the-art pneumonia classification with deep learning approaches has limitations for being lightweight, data-hungry, and lacking a global perspective, especially concerning the geometrical, orientational, and variational knowledge of inflammation within the CXR. Therefore, our research objective is simple: to develop a deep learning-based model to classify pneumonia inflammation within CXR with the property to understand a global perspective with underlying positional and geometrical transitions by the help of Capsule Networks Cluster and modified CAM for performing radiologist level of validation. The reason for using Capsule Networks Cluster is that they can understand geometrical and orientational details with images, which needs to be improved for CNN, GNN (graph neural network), GAN, and other Deep Learning Models today.

## 3 Materials

### 3.1 Dataset

**Fig. 3:**
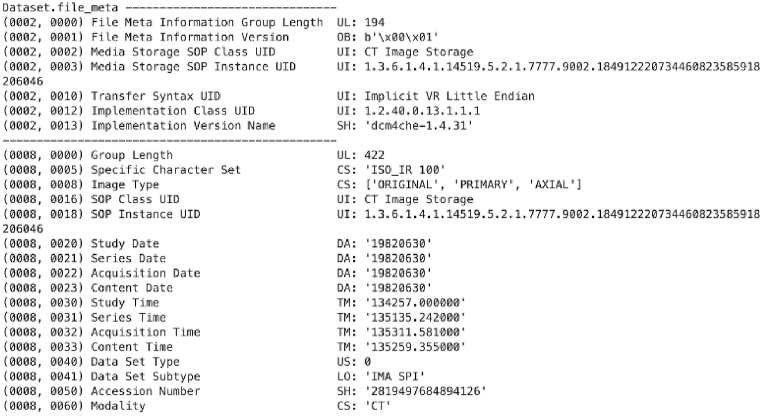
The Dicom file header output contains the Dicom file, imaging device, patient information, type of scan, body part, etc.

**Fig. 4:**
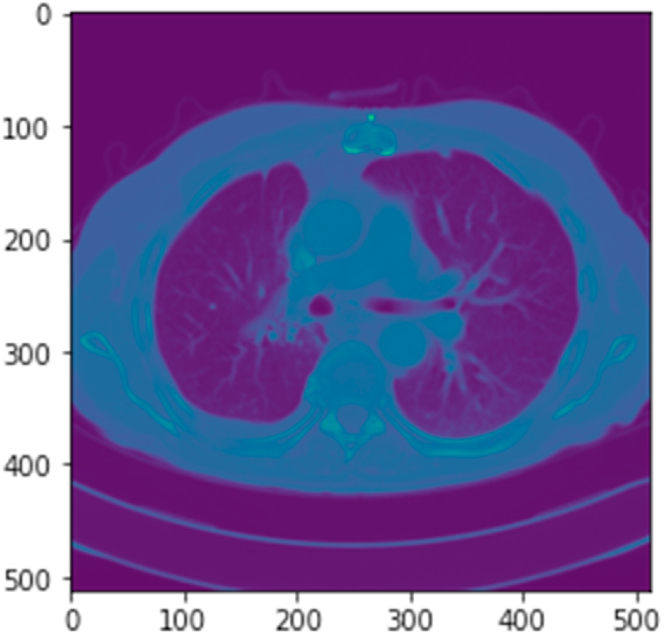
Dicom file body output containing an x-ray of the abdomen.

The Radiological Society of North America released a CXR dataset related to pneumonia inflammation in 2018. The dataset released contains approximately 26,684 files of CXRs in Dicom format. Of 26,684 images, 20,672 CXRs are without pneumonia, while 6012 CXRs are with pneumonia. That is much bias and a significant imbalance in the data. Generally, the Dicom format contains two main elements: (1). Header and (2) Body. Fig. 3 provides a snapshot of the Dicom file header output, while Fig. 4 provides the body output. The structure is as follows:

#### 3.1.1 Header

● Device Information (Modality/Scanner)
● Patient Information
● Study UID
● Series UID
● Image Transformation:
  1. Shape
  1. Slice
  1. Thickness
● etc.

#### 3.1.2 Body

● Image pixel data (2D, 3D, 4D, …)

The idea behind the release of the dataset is to put a stage of AI challenge over Google for prediction and classifying pneumonia within these with medical-grade accuracy. The Society for Thoracic Radiology and MD. ai have labeled the dataset. The National Institutes of Health gave chest X-rays, ensuring that all identifiable patient information is masked according to HIPAA compliance.

### 3.2 Preprocessing

The original size of CXR images is 1024x1024 pixels. Having this size requires extensive computation power with a good memory. Therefore, we reduced the size to 224x224 pixels during our proof of concept and training loops. The resizing allowed us to perform multiple training iterations and tuning within the limited resources and time constraints. Furthermore, we normalized the pixels from 0 to 1 to standardize the pixel diversity. The normalization process was instrumental during the learning phase, enabling our optimizer to navigate efficiently. For normalization, we employed the mean and standard deviation. Instead of the conventional method, which involves computing the mean and standard deviation for the entire dataset, a time-consuming and resource-intensive process, we calculated the sum and squared sum of each image's pixels individually in the dataset. Subsequently, these values were aggregated into global variables for the entire dataset, and the mean and standard deviation were computed using equations (1), (2)). Global statistical indices like mean and variance in image analysis can pose a challenge when dealing with localized pathologies. These statistics offer summary measures that describe properties of the entire image, encompassing both healthy and pathological regions, potentially missing the nuanced and localized variations in pixel intensities characteristic of specific pathologies. Pathologies often manifest as localized abnormalities or deviations from the norm, affecting specific image regions, including changes in pixel values, texture, or patterns. Global statistics like mean and variance amalgamate information from the entire image, possibly obscuring or diluting the local variations caused by pathologies. To address this, our research employs a capsule-based approach, where each capsule can accommodate a region of Hi x Wi (Height and width of input matrix representing features of x-ray scan for a capsule). The cluster of capsules collectively composes the entire image, facilitating the capture of region-specific mean and standard deviation values. This approach ensures that statistics are not diluted or neutralized due to global impact.

(1) μ=sums/N

(2) $$\sigma =\sqrt{\;}\left\{\left(\text{sumssquared}/N\right)\;-\;\mu^{2}\right\}$$

Here in equations (1), (2)):

● sums = represents the accumulated result of the individual pixels across all images in the dataset.
● sum squared = accumulated result of the squares of individual pixel values in the images
● μ = mean of the dataset
● σ = standard deviation of the dataset
● N = number of images of the training set

Equation (1) quantifies and understands the distribution of pixel values in a set of images. It provides insights into the dataset's central tendency (mean) and pixel values' spread (standard deviation). We used to characterize the overall intensity distribution and variability within the image dataset to get information about subsequent processing decisions for our capsnet cluster routing layers. The equations also help in generalizing the dataset for processing and analysis. The expression in equation (2) calculates the average of the squared pixel values, and subtracting μ^2^ ensures that we are measuring the variance from the mean. Taking the square root of this result provides the standard deviation, a measure of the dataset's spread or dispersion of pixel values. For our work, calculating the μ and σ of pixel values in the dataset is crucial for preprocessing. These metrics are vital in normalization, accelerating model convergence during training. The strategy assumes that the statistical attributes of pixel values (μ and σ) are stable across the broader dataset, reducing computational demands for calculating statistical metrics without compromising the presumed uniformity of pixel value characteristics throughout the dataset.

We used Z-normalization for our work and calculated using equation (3).

(3) $$x_{\text{norm}}=x\;-\;\mu /\sigma$$

● x = all pixel values.

We split the entire dataset into 3. (1). 75% for training, (2). 10% for validation and (3) 15% for testing. We split the dataset with a complete reshuffling to reduce the biases. However, it is worth mentioning that preprocessing is extensive work, and the final results depend a lot upon the preprocessing stage in any image classification job. We used a customized preprocessing methodology for our proposed capsule networks cluster. However, in general, we could always use fuzzy-based image association for a far more extensive preprocessing level, as done by Refs. [39,40]. Given the nature of our model in this research, our customized preprocessing can do the job effectively.

### 3.3 Interpretation

In our pursuit of creating a pneumonia classification system that aligns with the medical community's standards, we sought to bridge the gap between machine interpretation and how doctors approach the diagnosis. To achieve this, we implemented a modified version of Class Activation Maps (CAM), called mCAM [29], within our proposed work. Modified Class Activation Maps (mCAM) represent a noteworthy advancement in X-ray image analysis, particularly within the context of deep learning models. These maps, an extension of the conventional Class Activation Maps (CAM), hold significant promise in enhancing the interpretability and transparency of deep learning-based diagnostic systems in radiology. By tailoring the CAM approach to produce class-specific activation maps for distinct features in X-ray images, mCAM furnishes researchers with valuable insights into the model's decision-making process. This enhancement facilitates a deeper understanding of the critical factors influencing diagnostic outcomes, thus improving both the clinical utility and trustworthiness of AI-assisted diagnostic systems, which are pivotal for accurate patient care. In our work, the mCAM methodology effectively fences off regions in chest X-rays with the most pronounced signs of inflammation, mimicking the visual cues that radiologists and physicians rely on during image inspection. This approach mirrors the diagnostic process where medical professionals compare the areas of inflammation with non-inflamed regions within the X-rays, calculating the percentage to determine the likelihood of pneumonia. Furthermore, the use of class activation maps aids in discerning the localization of discriminative features within the deep layers of the model, thereby enhancing our understanding of the network's decision-making process and its clinical relevance.

The calculation of the class activation map with a modified approach to handling a cluster of inflammation capsule networks is given in Equation (4) [29].

(4) $$M=\surd^{n}{\sum^{n}\;}_{i=1}\left\{{\sum^{m}\;}_{k=1}\left\{\left(w_{k}.c\right)*A\right\}\right\}$$

Equation (4) is a representation of Modified Class Activation Maps (mCAM), which is used in the context of our capsule networks cluster for visualizing the regions of importance within an image that contribute to a pneumonia classification by a capsule.

Here's a breakdown of the equation:

● M = represents the class activation map for an image's feature of interest in our case, “pneumonia inflammation”
● n = signifies the number of features for visualization.
● The outer summation symbol "∑" represents the summation across all features coming from each capsule of the cluster.
● i = counter for the cluster of inflammation capsule
● The inner summation "∑" represents the summation within a class or feature.
● k = spatial/location filters on the inflammation layer, which can be acquired through the capsule network
● w_k_ = weights of k
● c = denotes feature-specific information in the network as routing coefficient
● A = feature maps or activations at a specific layer of the network.

In the context of our work, mCAM is used to compute feature-specific activation maps to visualize which regions or features in an image were particularly relevant for the network to make a certain classification of pneumonia inflammation. It helps enhance the interpretability of capsule network clusters by highlighting the regions that contributed the most to the network's decision for the inflammation feature.

The mCAM equation is a pivotal component of our methodology, delineating the output, or features, of the final convolution layer within our model. This process plays a crucial role in defining the specific region in a chest X-ray (CXR) image that exhibits the highest likelihood of inflammation. The coupling coefficient is an integral aspect of the capsule network, providing a means for efficient backward propagation. It ensures that only features with meaningful relationships from previous layers, denoted as “i" layers, are included in the computational process. This selective approach filters out unrelated features, thus reducing invariance and enhancing the covariance among the extracted features.

Moreover, our methodology incorporates a normalization factor for the classification activation maps. This factor is derived from the sum of the square roots of the entire dot product involved in the computation. Its function is to ensure that only those regions demonstrating high likelihoods of inflammation are retained, while those that could potentially produce false-positive results are eliminated. In this way, our model seeks to provide a refined and accurate identification of regions in CXR images exhibiting signs of inflammation while minimizing the risk of misleading or inaccurate outcomes. This novel combination and alignment with medical-grade diagnosis have yet to be tried in published research.

Here in Fig. 5:

● H_i_ & W_i_: Height & Width of the n-dimensional matrix representing X-ray scan
● D_pc_: Dimensions of Primary capsule
● C_n_: Input Channel of X-ray scan
● C_o_: Output Channel of X-ray scan
● K_h_: height of the kernel

**Fig. 5:**
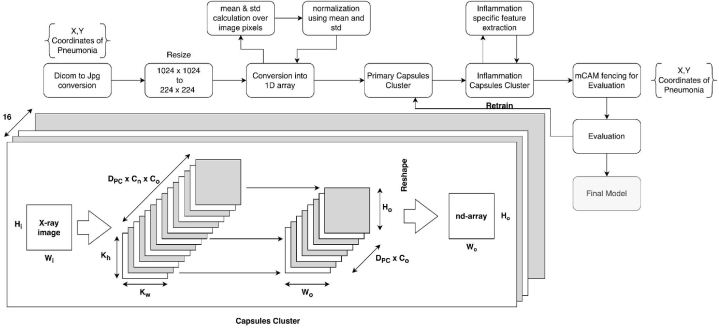
Block diagram of complete training methodology of our proposed methodology.

## 4 Methodology

Our approach leverages a pre-trained Capsule Networks Cluster [28], initially trained on the Flickr dataset for generating image captions with detailed positional and geometrical information. We have customized this pre-trained model to extract inflammation-specific features within the inflammation capsule layer. In contrast to the original model, we have adjusted the primary capsule layer to generate eight feature outputs instead of 16, and we have adapted the input layer to process single-channel images instead of the usual three channels. The output from the inflammation capsule is then directed into mCAM-based fencing, which generates class-activated maps, highlighting regions of the image most likely to contain inflammation features with the highest probability. This model modification aims to enhance its performance in identifying inflammation-related patterns.

Our research methodology encompasses a multi-step process designed for robust pneumonia classification. Beginning with the intake of Dicom files, our system initially transforms them into image and data blocks. The image blocks are then converted into n-dimensional arrays, facilitating the calculation of mean and standard deviation, as elaborated in Section 3.2. Subsequently, the blocks are normalized using these statistics and stored in another n-dimensional array. This array is the input to our primary capsule layer. The primary capsule layer, integral to our feature extraction mechanism, carries out convolution and routing by agreement over the data, yielding an 8-feature block specific to inflammation. The inflammation layer extends these 8-feature inputs from the primary capsule layer into two analogous 8-feature blocks, which are then directed to a 16-capsule cluster within the inflammation layer. Here, extracted features undergo feature-specific extraction as part of the inflammation layer's process.

● K_w_: width of the kernel
● H_o_ & W_o_: Height & Width of output matrix representing features of x-ray scan.

These features are subsequently channeled to class activation blocks, enabling the delineation of regions indicative of inflammation. The model compares these fencing regions to the x and y coordinates of the Dicom files representing inflammation in the X-ray scan. When a strong correlation exists between the fencing region and the actual inflammation site, the model outputs a probability of pneumonia for the respective patient. To bolster the model's robustness, we validate its predictions by comparing them with actual image labels during the training and tuning phases. For a visual representation of our model's execution cycle for pneumonia classification, please refer to Fig. 5. Our primary capsule cluster serves as the primary feature extraction engine, providing critical features to the inflammation capsule cluster for job-specific feature extraction and feature flattening.

Here in Fig. 6:

● H_o_ & W_o_: Height & Width of the n-dimensional matrix representing the output of primary caps layer
● n: number of layers
● FC_n_1: FC layer 1
● FC_n_2: FC layer 2
● FC_n_3: FC layer 3

**Fig. 6:**
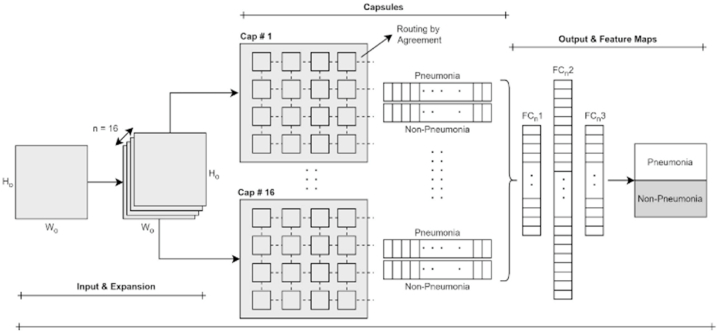
Block diagram of the complete inflammation layer of our proposed methodology.

The routing by agreement stage in all preceding layers facilitates the selective advancement of features with relevant relationships, discarding features lacking meaningful connections. This approach underscores a more connection-centric feature extraction process than traditional CNN models, which extract features independently of their relational significance. A detailed depiction of the inflammation capsule cluster, central to our research, can be found in Fig. 6.

The inflammation capsule layer performs convolution and then routing by agreement with the input data from the primary capsule cluster layer. The output feature maps are then to the FC layers (FC_n_1, FC_n_2 & FC_n_3). Finally, the FC_n_3 layer flattens the data and uses it to compare the actual data. The novelty in our work is that the comparison is not only done based on labels of original data; the comparison also uses modified class activation maps, which help classify the outcomes from the perspective of the medical side.

During the training and evaluation phase, we used an ADAM optimizer with a learning rate of 1e^−4^. The loss function is BCEWithLogitsLoss. The total training epochs were 100, and the batch size was 64. The splits were 75%, 10%, and 15% on the random data shuffling. The training time is given in Equation (9) and represented as TToT. The total trainable parameters during the training were 18.2 million, and the total model parameters' size in memory was 128.63 MB.

### 4.1 Evaluation

Along with the modified Class Activation Maps to evaluate our model performance for the classification of Pneumonia within the CXRs, we have also used standard metrics to gauge the overall performance. The metrics are accuracy, recall (sensitivity), F1-score, and confusion matrix. The equations for standard metrics are given in equations (5), (6), (7), (8).

(5) $$\text{Accuracy}=\left(T_{P}+T_{N}\right)\;/\;\left(T_{P}+T_{N\;}+F_{P}+F_{N}\right)$$

(6) $$\text{Recall}\;\left(\text{Sensitivity}\right)=T_{P}\;/\;\left(T_{P}+F_{N}\right)$$

(7) $$\text{Precision}=T_{P}\;/\;\left(T_{P}+F_{P}\right)$$

(8) $$F1_{\text{score}\;}=2\;x\;\left\{\left(\text{Precision}*\text{Recall}\right)\;/\;\left(\text{Precision}+\text{Recall}\right)\right\}$$

Here in equations (5), (6), (7), (8):

● T_P_: True Positive predictions
● F_P_: False Positive predictions
● T_N_: True Negative predictions
● F_N_: False Negative predictions

The entire set of results is measured based on the above-given metrics. However, to get a more exciting understanding of the model, we measured the feature extraction time in primary caps and inflammation caps layers, the total training time of the model, and the time to generate a response on testing data. These measurements helped understand the model efficiency from the perspective of real-world implementations.

The total time consumed for the training can be seen in eq. (9). The total time of execution for the training of our model is 1780 s, and the saving of the model takes approximately 20 s:

(9) $$T_{\text{ToT}}=e_{t}\;*\;t_{e}+m_{\text{st}\;}$$

Here in eq. (9):

● T_ToT_ = Total Time of Training
● e_t_ = 1 epoch time (17.8 s)
● t_e_ = total epochs of training (100)
● m_st_ = model saving time (20) seconds

The computational complexity of Capsule Networks (CapsNets) depends upon factors like Number of Capsules, Capsule Dimension, Routing Iterations, Matrix Multiplication, Dynamic Routing, Activation Functions, Training Data Size, and Batch Size. Capsule Networks offer advantages in capturing hierarchical relationships in data. They can be computationally demanding, particularly when dealing with large datasets and complex network configurations. In our research, the complexity is controlled by the number of capsules in our cluster, activation function, and the iterations to have a balanced approach to reaching the results. The computational complexity of a single capsule's forward pass operation can be calculated using formula O (k.d.m). The elements of the formula are as follows:

● O = represents the order of complexity.
● k = the count of capsules in the current layer.
● d = the dimensionality of the capsule output vectors.
● m = the number of capsules in the subsequent layer.

formula O (k.d.m) explains the complexity of computing the output of a capsule in terms of the number of capsules in both the current and subsequent layers and the dimensionality of the capsule vectors. However, the complexity of the capsule networks cluster depends upon the number of capsules and operations being performed in capsules. The formula for the complexity of the capsule networks cluster can be written as O(N⋅d⋅R⋅Complexity of Capsule Operations). The elements of the formula are as follows:

● N = the number of capsules in the cluster
● R = Number of Routing Iterations

## 5 Experimentation & results

The experimental setup for our work comprises a 32-core Xeon processor, 64 GB of RAM, and 8 GB of Nvidia GeForce 1080. The original image acquired from Dicom files is resized to 224 × 224, earlier 1024 × 1024. The image resizing was done with the assurance that the cropping would not affect the inflammation coordinates in the image. Table 1 shows the class distribution of chest X-rays for pneumonia and non-pneumonia for training, validation, and testing split.

**Table 1:** Distribution of normal and pneumonia classes with training, validation & testing datasets.

S#	Class	Training Set	Validation Set	Testing Set
**1**	Pneumonia	4400	732	880
**2**	Normal	15600	2002	3070
**3**	Total	20000	2734	3950

**Fig. 7:**
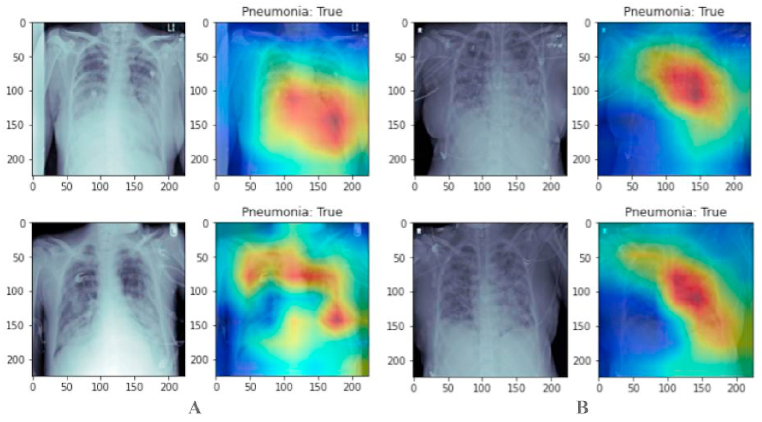
Classification result of chest x-rays for positive pneumonia using our capsule networks cluster model. In Panel “A” the right side of images are original while the left sides of images are processed through our model. In Panel “B” the right side of images are original from data side while the left side shows detected penuomenia in them.

**Fig. 8:**
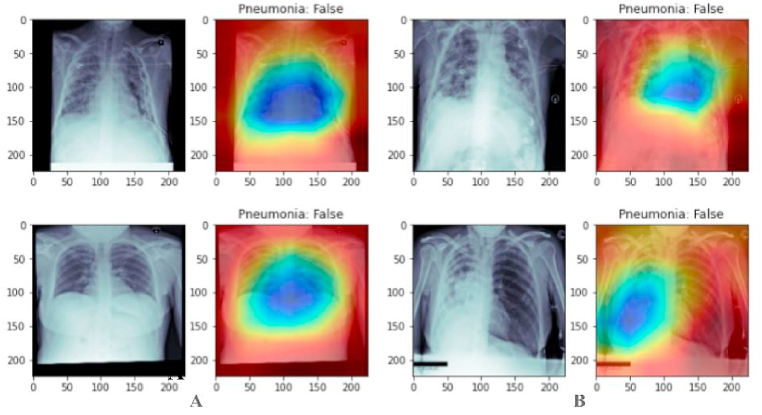
Classification result of chest X-rays for negative pneumonia using our capsule networks cluster model. In Pnaels “A” and “B” the right sides of images are the original image while the left sides of the images shows the unavailability of pneumonia with them as detected by our model.

The image group in Fig. 7 provides a graphical interpretation of positive pneumonia detection results during pneumonia classification over chest X-rays. In contrast, the image group in Fig. 8 provides an understanding of negative pneumonia detection results. The images are taken from the test dataset and have never been shown to the model earlier.

Table 2 provides the details of parameters and the accuracy as the comparison between our approach and other state-of-the-art research works in the domain. The modeling parameters, being huge in numbers, have been trained with minimal time as the total training epochs were 100, and each epoch took approx. 17.8 s to complete the entire training dataset over the batch size of 64 images per batch.

**Table 2:** Comparison of acquired accuracy and training parameters.

S#	Model	Type	Params	Accuracy
**1**	**CapsNet Cluster**	**CapsNet**	**18.2 M**	**98.3%**
**2**	COVID-Net [32]	DCNN	11.75 M	93.3%
**3**	CNN [31]	VGG16	13.8 M	94.5%
**4**	VGG16 [30]	NN with VGG16	NA	95.4%

Table 3 details the training and validation metrics: accuracy, precision, recall, and F1-score, along with the confusion matrix over validation data in Table 4. The confusion matrix is generated for 2668 images from the validation dataset. The mCAM function generates medical-specific patterns for visualizing pneumonia in chest X-ray scans by reshaping the output tensor into 512 × 49 tensors for a simple multiplication factor. Then, the feature matrix is reduced to weights only by removing the bias. Next, the weight and features are computed with the equation given in 4, and then the square root accumulation of all individual results is performed. Later, the values are normalized and sent back to the CPU for visualization. The result of the mCAM function output is given in Fig. 7, Fig. 8.

**Table 3:** Details about the metrics of training and validation.

S#	Metric	Training	Validation
**1**	Accuracy	0.962	0.960
**2**	Precision	0.921	0.920
**3**	Recall	0.893	0.889
**4**	F1-Score	0.906	0.913

**Table 4:** Result of Confusion Matrix over validation dataset.

	T_P_	T_N_
**F**_**P**_	2260	70
**F**_**N**_	57	347

Table 5 provides a comparative analysis of accuracies acquired by our approach compared to others concerning the size of the dataset used for the research. Generally, accuracies are meant to drop with the data size, while in our case, the dataset size is transparent for the model. Hence, the accuracy is high compared to the others despite using a small dataset for training and validation.

**Table 5:** Comparison of Accuracies with literature review articles.

S#	Paper	Accuracy	Dataset Size
**1**	51	98.6%	5856
**2**	52	96.81%	6118
**3**	53	98.4%	5856
**4**	Ours	98.3%	26,684

## 6 Conclusion

The earlier the problem is detected, the better the solution, which is the essence of our research. The early detection of pneumonia with medical-grade accuracy just by CXRs can help solve the issue before it worsens for patients. Our lightweight and industry-applicable model, depending upon the capsule networks cluster, provides more than 96% accuracy in classifying the patient case and the modified CAM, giving actual coordinates of inflammation within the CXRs. The acquired F1-score of approx. 91% also symbolizes the acceptance of classification jobs within the healthcare industry. Understanding global perspective and training for small data understanding multiple variations has helped us reach a relatively sound and industry-oriented research objective. We evaluated many CXRs from other sources and found the result promising and up to healthcare industry standards. In the future, we would like to improve the detection time and make a complete cloud-based pipeline for doctors and radiologists to test the model globally.

### 6.1 Limitation of the proposed research

Our methodology covers a generalized area of medical imaging for CXR. It can be used in real-time without extensive training time and massive datasets. The combination of Capsule Networks Cluster and mCAM enables the methodology to be medical-centric instead of deep learning-centric. However, the technique is relatively new, especially since there is not much research on capsule networks, nor are any already trained models available, so reaching the working system requires training the model from scratch. The other limitation that can be slightly significant is integrating the proposed technique directly with different vendor X-ray machines. The methodology can directly digest Dicom files, but the interfacing with the machine is subject to the available interfacing protocol. Color Jitter, contrast-limited Adaptive Histogram Equalization, and Composite Transformations were not tested during the study for the pneumonia-related CXR analysis. This can also be treated as a limitation; however, all three generally apply to multicolor and multi-object images.

## Data availability

The dataset used for our research's training, validation, and testing was taken from the Radiological Society of North America.

(RSNA). The dataset version is 2018, and it is available publicly for research on the below-given links:
● https://s3.amazonaws.com/east1.public.rsna.org/AI/2018/pneumonia-challenge-dataset-original2018.zip
● https://s3.amazonaws.com/east1.public.rsna.org/AI/2018/pneumonia-challenge-annotations-original2018.json
● https://s3.amazonaws.com/east1.public.rsna.org/AI/2018/pneumonia-challenge-dataset-mappings2018.json

1. Has data associated with your study been deposited into a publicly available repository?

The data is publicly available and can be downloaded from the above URLs.

AN(s), JN/BT, chapter title/article title, year of publication, volume number/book chapter, pagination and the DOI should be included.

## CRediT authorship contribution statement

**Anwar ul Haque:** Writing – original draft. **Sayeed Ghani:** Writing – review & editing, Supervision, Formal analysis, Conceptualization. **Muhammad Saeed:** Writing – review & editing, Validation, Investigation, Data curation. **Hardy Schloer:** Writing – review & editing, Supervision, Resources.

## Declaration of competing interest

The authors declare that they have no known competing financial interests or personal relationships that could have appeared to influence the work reported in this paper.
